# Increased Turnover of Dopamine in Caudate Nucleus of Detoxified Alcoholic Patients

**DOI:** 10.1371/journal.pone.0073903

**Published:** 2013-09-11

**Authors:** Yoshitaka Kumakura, Albert Gjedde, Daniele Caprioli, Thorsten Kienast, Anne Beck, Michail Plotkin, Florian Schlagenhauf, Ingo Vernaleken, Gerhard Gründer, Peter Bartenstein, Andreas Heinz, Paul Cumming

**Affiliations:** 1 Department of Neuroscience and Pharmacology, University of Copenhagen, Copenhagen, Denmark; 2 Center for Functionally Integrative Neuroscience, Aarhus University, Aarhus C, Denmark; 3 Department of Nuclear Medicine, Tokyo University, Tokyo, Japan; 4 Department of Experimental Psychology, Cambridge University, Cambridge, United Kingdom; 5 Department of Psychiatry, Charité - Universitätsmedizin Berlin, Berlin, Germany; 6 Department of Nuclear Medicine, Charite - Universitätsmedizin Berlin, Berlin, Germany; 7 Department of Psychiatry, University of Aachen, Aachen, Germany; 8 Department of Nuclear Medicine, Ludwig Maximilian University of Munich, Munich, Germany; University of Manchester, United Kingdom

## Abstract

A previous study of the DOPA decarboxylase substrate 6-[^18^F]fluoro-*L*-DOPA (FDOPA) with positron emission tomography (PET) detected no difference of the net blood-brain transfer rate (*K_in_^app^*) between detoxified alcoholic patients and healthy controls. Instead, the study revealed an inverse correlation between *K_in_^app^* in left ventral striatum and alcohol craving scores. To resolve the influx and efflux phases of radiolabeled molecules, we independently estimated the unidirectional blood-brain FDOPA clearance rate (*K*) and the washout rate of [^18^F]fluorodopamine and its deaminated metabolites (*k*_loss_), and we also calculated the total distribution volume of decarboxylated metabolites and unmetabolized FDOPA as a steady-state index of the dopamine storage capacity (*V_d_*) in brain. The craving scores in the 12 alcoholics correlated positively with the rate of loss (*k_loss_*) in the left ventral striatum. We conclude that craving is most pronounced in the individuals with relatively rapid dopamine turnover in the left ventral striatum. The blood-brain clearance rate (*K*), corrected for subsequent loss of radiolabeled molecules from brain, was completely normal throughout the brain of the alcoholics, in whom the volume of distribution (*V_d_*) was found to be significantly lower in the left caudate nucleus. The magnitude of *V_d_* in the left caudate head was reduced by 43% relative to the 16 controls, consistent with a 58% increase of *k_loss_*. We interpret the findings as indicating that a trait for rapid dopamine turnover in the ventral striatum subserves craving and reward-dependence, leading to an acquired state of increased dopamine turnover in the dorsal striatum of detoxified alcoholic patients.

## Introduction

In non-alcoholic drinkers, alcohol intake evokes acute release of dopamine in ventral striatum, as revealed in vivo by reduction of dopamine D_2/3_ receptor availability [1]. Repeated exposures to alcohol subsequently alter the phasic and tonic dynamics of dopamine release in rodent striatum [2]–[4] and therefore predict similar changes of dopamine markers in patients with chronic alcohol abuse. In detoxified alcoholic patients, the competition between endogenous dopamine and exogenous tracer [^11^C]raclopride, estimated by means of positron emission tomography (PET), reveals attenuation of the dopamine release evoked by methylphenidate [5] or amphetamine [6]. These observations raise the question of changes in the dopamine synthesis capacity, which is one of the most robust indicators of psychotic states [7]. In one PET study of alcoholism, detoxified alcoholic patients had significantly elevated influx of the DOPA decarboxylase substrate 6-[^18^F]fluoro-*L*-DOPA (FDOPA) to left putamen and right caudate nucleus [8], while in a subsequent study, detoxified alcoholic patients had no change of FDOPA uptake [9], although a correlation was seen with alcohol craving.

The measure of the net blood-brain transfer rate of FDOPA obtained in these studies of alcoholic patients (*K*_in_^app^; ml g^−1^ min^−1^) reflects both formation of [^18^F]fluorodopamine and the subsequent washout of diffusible acidic metabolites [10], which are known to respond differentially to changes of trait or state markers of dopaminergic neurotransmission [11]–[16], as shown previously [17], [18]. The analysis separating synthesis from loss in the dopamine metabolic pathway yields the unidirectional clearance rate of FDOPA to brain tissue by enzymatic actions (*K*; ml g^−1^ min^−1^) and the fractional rate constant for elimination of [^18^F]fluorodopamine and its metabolites (*k_loss_*; min^−1^). The ratio between the enzymatic clearance of FDOPA (*K*) and the rate of loss (*k_loss_*) constitutes a steady-state volume of distribution for the decarboxylated and deaminated metabolites, which reflects the total distribution volume in brain (*V_d_*; ml g^−1^), an index of dopamine storage capacity, as detailed in the earlier publications [19]–[22].

We predicted that the previous finding of an inverse correlation between the net blood-brain transfer (*K*_in_^app^) and alcohol craving scores [23] was derived from trait-dependent differences of the catabolism of [^18^F]fluoro-dopamine formed in ventral striatum and subsequent washout of the breakdown products [24]. Here, we tested the hypotheses, first, that the individual rates of [^18^F]fluorodopamine washout (*k_loss_*) in the left ventral striatum would correlate with the severity of craving in the group of alcoholics, as previously noted for the net blood-brain transfer (*K*_in_^app^), and second, that the analysis would reveal increased *k_loss_* throughout striatum of detoxified alcoholic patients, relative to an age-matched control group.

## Materials and Methods

### Subject Recruitment

All subjects provided written informed consent for participation in the studies which had been approved by the local Research and Ethics Committees of both universities in Denmark and Germany (De Videnskabsetiske Komitéer for Region Midtjylland, Ethikkommission der Landesärztekammer Rheinland Pfalz). The control group consisted of the 12 volunteers from the earlier study [23], augmented with four additional subjects from other studies [22], [25], [26], giving a total of 16 healthy right-handed male subjects aged 32–55 years (mean 43.2±8.1 years). Twelve right-handed detoxified male patients with alcoholism aged 32–57 years (mean 42.5±7.5 years) were the patient cohort in the previously-published FDOPA PET study [23]. The healthy control subjects had no psychiatric axis I or II disorders, according to assessment with the SCID [27], [28]. All control subjects were non-smokers. The patients met the ICD-10 and DSM-IV criteria for alcohol dependence and had no other axis I psychiatric disorders and no past history of drug dependence or current drug abuse, according to assessment with random urine and breath tests in a supervised inpatient treatment program for a mean of 36 days (SD 22) prior to scanning [9].

The mean (± SD) alcohol lifetime alcohol consumption of the patients was 1038±828 kg, and they smoked a mean (± SD) of 17±13 cigarettes per day, yielding a weak trend towards correlation between individual alcohol and cigarette consumption (r = 0.27; n.s.). The lifetime alcohol intake was measured with the Lifetime Drinking History questionnaire [29]. The severity of current alcohol craving was measured with the Alcohol Craving Questionnaire (ACQ: [30]) on the morning before FDOPA PET scanning. The ACQ is a widely and internationally used instrument with good test-retest reliability (kappa = 0.85, p<0.001; tested in 46 alcoholic subjects on two separate days) and high internal consistency (Cronbach’s alpha = 0.96, p<0.001, N = 243). Daily smoking habits reported by the patients and control subjects were recorded, and treated as a nuisance parameter in statistical analyses for the voxelwise group comparisons and correlations with ACQ scores.

### PET Scanning and Plasma Sampling Procedure

Subjects fasted overnight, and received carbidopa (Merck Sharpe and Dohme; 2 or 2.5 mg/kg, p.o.) one hour prior to the PET scan in order to minimize the decarboxylation of FDOPA in peripheral tissues [31]. Dynamic attenuation-corrected emission images were acquired with ECAT EXACT 47 whole body PET scanners (CTI/Siemens, Knoxville, TN), as described in detail in the earlier report [23] during 120 minutes following intravenous injection of FDOPA (200 MBq). Total radioactivity concentration in serial samples of arterial blood was measured in well-counters cross-calibrated to the tomographs. The fractions of untransformed FDOPA and its major plasma metabolite 3-O-methyl-6-[^18^F]fluoro-*L*-DOPA (OMFD) were determined in selected samples by reverse-phase high performance liquid chromatography [31]. Continuous plasma input functions for FDOPA and OMFD were calculated by fitting bi-exponential functions to the measured fractions [32].

In the present study, dynamic PET sequences were realigned and corrected frame-wise for head motion, and then summed and registered to the Montreal Neurological Institute (MNI) stereotaxic brain, as described previously [19], [25]. In short, we created a tissue image, which represented mean radioactivity distribution averaged over the time period of 20–120 min, after preparatory frame-wise head motion correction. The FDOPA tissue image then underwent linear transformation to the MRI gray matter template image of the MNI space, which was modified for voxel intensity distribution to mimic FDOPA accumulation of striatum and cerebral cortex. In the process of transformation to the MNI space, the linear transformation algorithm was weighted more towards striatum than cerebral cortex contour. This weighting also minimizes the potential atrophy effect in striatum.

Sixty minute cerebellum TACs were first analyzed using a constrained compartment model with dual arterial inputs for OMFD and FDOPA [10], [13]. The VOI template of cerebellum had a sufficiently large volume of 48 cm3 to minimize partial volume effects related to potential atrophy. The OMFD concentration curve in brain calculated relative to the entire 120 min OMFD input was then globally subtracted from the entire dynamic emission sequence [21]. Briefly, we first estimated kinetic parameters for OMFD, and then precisely identifid the OMFD accumulation in cerebellum, using the basic *K_1_-k_2_* neurokinetic model. The OMFD curve was regenerated mathematically for subtraction of OMFD contamination. This OMFD subtraction method permits the two hour tomography, which is sufficient for subsequent parameter estimation of the present study. Following this mathematical subtraction of brain OMFD, FDOPA kinetics in brain simplifies to an “inlet-outlet” model [17], , comprising the intrinsic blood-brain clearance rate of FDOPA (*K*, ml g^−1^ min^−1^), and the fractional rate constant for the process of elimination of [^18^F]fluorodopamine together with its deaminated metabolites from brain (*k_loss_*, min^−1^). The ratio (*K*/*k_loss_*) is analogous to an effective distribution volume (*EDV* ml g^−1^), as defined in [15]. In the method used here, the steady-state total tracer distribution volume in brain (*V_d_*, ml g^−1^) equals the sum of the FDOPA plasma volume (*V_0_*, ml g^−1^), the distribution volume of unmetabolized FDOPA in brain (*V_f_*, ml g^−1^), and the decarboxylated metabolite pool, consisting of [^18^F]fluorodopamine and its deaminated metabolites (*K*/*k_loss_* ml g^−1^; see [19], Fig. 2C). Robust estimates of this triad of kinetic parameters are obtained through a multi-linear solution of a set of first-order differential equations, using the OMFD-subtracted brain TACs in the interval from 20 to 120 min [17], [20].

To search for brain regions with perturbed [^18^F]fluorodopamine kinetics, we calculated parametric *V_d_* maps in the native PET space using MATLAB (The Mathworks, Natick, MA). In the common MNI stereotaxic space after anatomical standardization, mean *V_d_* maps were calculated for the two groups, and difference maps were calculated by subtraction. Voxel-wise statistical analysis was performed using the general linear model (GLM), in order to search for cluster volumes and partial out the effects of smoking rates. Group differences and voxel-wise regression of *V_d_* with ACQ scores for the 12 patients were calculated using the GLM implemented in *Glim Image*, a program developed at the MNI. Then, cluster VOIs were defined directly from the raw t-maps, where the t-value threshold was set arbitrary to form a cluster (t>2.5–2.8), but as high as possible to avoid false positive voxels. The cluster VOIs identified in the *V_d_* voxelwise contrast and regression analyses were applied to the OMFD-subtracted dynamic 4D recordings for TAC extraction, and the individual magnitudes of *K*, *k_loss_* and *V_d_* were calculated separately from the TACs. The kinetic estimates obtained from the cluster VOIs were tested using the GLM analysis for the contrast between alcoholic and control groups as well as the regression with ACQ scores, treating individual smoking rates as a nuisance variable.

## Results

**Figure 1:**
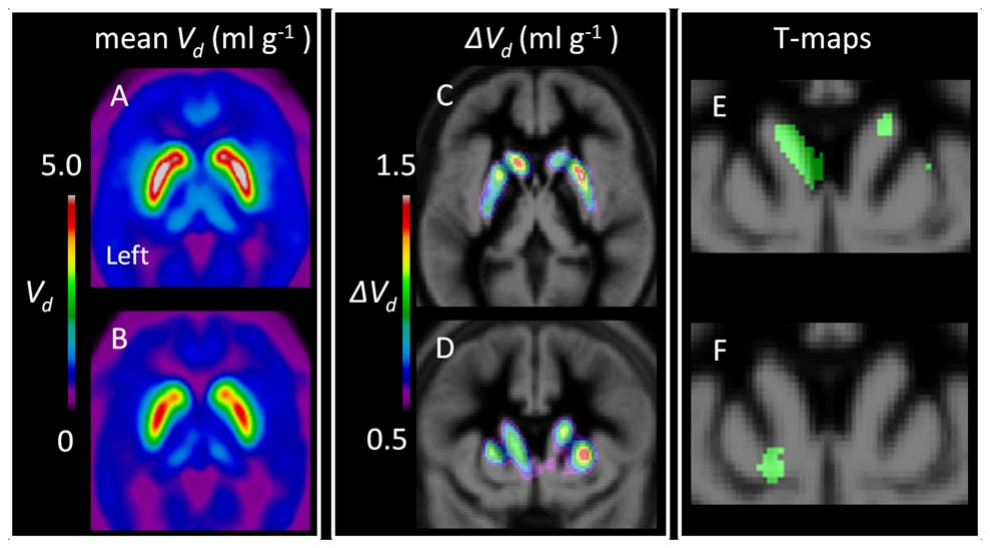
The parametric maps show mean steady-state storage capacity for FDOPA (*V_d_*; ml g^−1^) in age-matched control subjects (A) and in abstinent patients with alcoholism (B), together with the subtraction map in horizontal (C) and coronal (D) planes projected onto the MNI brain atlas. The t-maps show clusters of voxels with t >2.8 (E: green area) for the contrast between patients (N = 12) and age-matched healthy control subjects (N = 16), and the cluster of voxels with t >2.5 (F: green area) for the correlation between FDOPA-*V_d_* and the individual score in the ACQ questionnaire of craving in the alcoholic group, accounting for the effect of the nuisance covariate (smoking).

On average, the group of detoxified alcoholics had reduced [^18^F]fluorodopamine storage capacity, as revealed by both the mean parametric *V_d_* map (Figure 1A,B) and the subtraction map (Figure 1C,D). The voxelwise comparison revealed a large cluster (t >2.8) in the left medial caudate nucleus where the mean magnitude of *V_d_* was lower in the alcoholic group (Figure 1E). Within this cluster, the mean magnitudes of *K* did not differ significantly between the groups, whereas in the group of alcoholics the mean magnitude of *k_loss_* was greater by 58% (P = 0.0003) and the mean magnitude of *V_d_* was reduced by 43% (P = 0.002) (Table 1); there were no significant correlations between the triad of FDOPA kinetic parameters and the ACQ scores.

**Figure 2:**
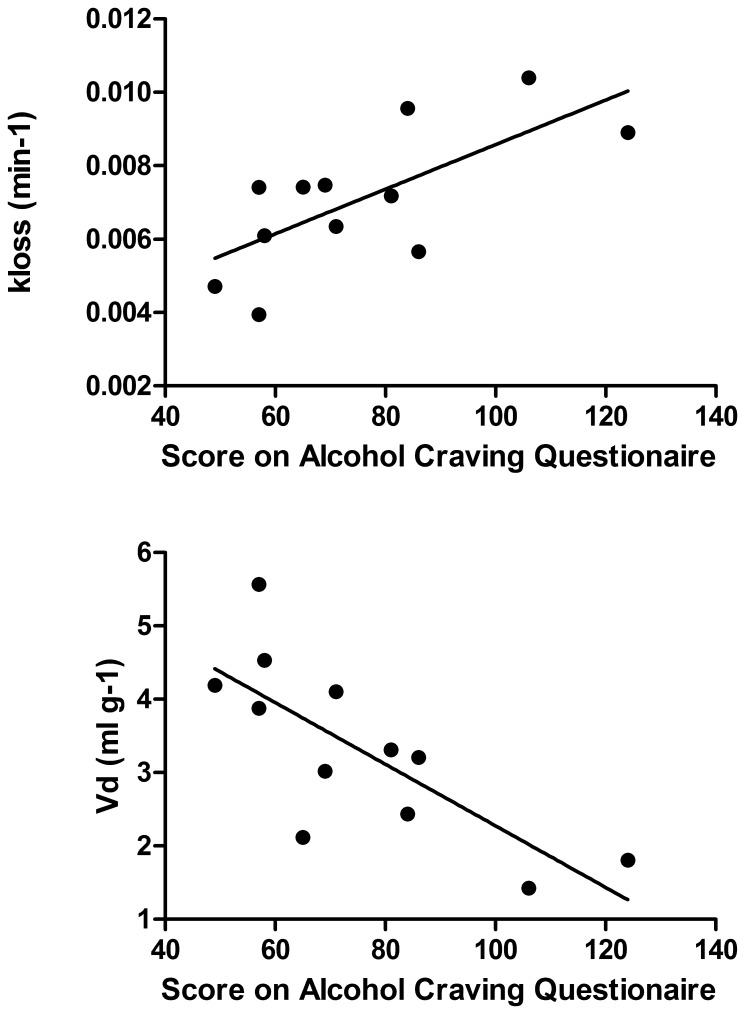
The scatter plots show the correlation between acute alcohol craving and the magnitude of the fractional rate constant for elimination of [18F]-fluorodopamine formed in brain (upper: *k_loss_*, min^−1^), and steady-state storage of FDOPA and its decarboxylated metabolites (lower: *V_d_*, ml g^−1^) for the left ventral striatum cluster. The lines indicate simple linear regressions in these scatter plots.

**Table 1:** The upper and lower tables present mean kinetic estimates of the voxel clusters identified by parametric mapping analysis.

Lt CDH	Controls(n = 16)	Detoxified Alcoholics(n = 12)	B (withACQ)	B (cigarettes/day)
*K*	0.0170±0.0032	0.0153±0.0039	n.s.	n.s.
*k_loss_*	0.0078±0.0023	0.0123±0.0039*** (+58%)	n.s.	n.s.
*V_d_*	2.36±0.77	1.36±0.54** (−43%)	n.s.	n.s.
**Lt VST**	**Controls (n = 16)**	**Detoxified Alcoholics (n = 12)**	**B** **(with** **ACQ)**	**B (cigarettes** **/day)**
*K*	0.0235±0.0045	0.0213±0.0045	n.s.	n.s.
*k_loss_*	0.0082±0.0022	0.0071±0.0018	0.773^†^ (R: 0.725)	n.s.
*V_d_*	3.00±0.88	3.30±1.17	−0.835^††^ (R: 0.782)	n.s.

The cluster of left medial caudate head (Lt CDH) was identified in the contrast between the alcoholic and healthy groups, whereas the left ventral striatum cluster (Lt VST) was identified by voxel-wise multivariate regression analysis with the individual ACQ scores of the patient group, partialling out the effects of cigarette consumption (numbers of cigarettes smoked per day as a nuisance covariate). The kinetic parameters are the blood-brain FDOPA clearance corrected for loss of trapped metabolites (*K*, ml g^−1^ min^−1^), the fractional rate constant for elimination of [^18^F]-fluorodopamine formed in brain (*k_loss_*, min^−1^), and steady-state storage of FDOPA and its decarboxylated metabolites (*V_d_*, ml g^−1^), as calculated using the linear solution of Equation 8 in [17]. Each estimate is the mean ± SD of 16 normal controls or 12 patients. P values were calculated using GLM. Significance of difference between alcoholic and control groups: (**) P<0.005; (***) P<0.001. Standardized partial regression coefficients (β) and multiple correlation coefficients (R) are presented for significant correlation between kinetic results in patients and ACQ scores: (^†^) P<0.05; (^††^) P<0.005. Results uncorrected for multiple comparisons.

Within the alcoholic group, voxelwise regression of the magnitude of *V_d_* as a function of the ACQ scores revealed a cluster (t >2.5) with inverse correlation in the left ventral striatum (Figure 1F). In this cluster, the magnitudes of *K* did not correlate with the ACQ scores. The magnitudes of *V_d_* correlated inversely with the ACQ scores, whereas the magnitudes of *k* ***_loss_*** correlated positively with the ACQ scores, with individual smoking rates treated as a nuisance parameter in the regressions (Table 1); there were no significant group differences in any of the triad of FDOPA parameters within the left ventral striatum cluster. Exclusion of smoking rate from the GLM analysis did not substantially alter the results, as seen in the simple plots of Figure 2 (statistical values not shown).

## Discussion

Two previous PET studies of dopamine synthesis capacity with FDOPA in alcoholic patients yielded partially disparate results [8]. In the latter study [23], the linear graphical analysis of the net blood-brain transfer rate of FDOPA to brain (*K_in_^app^*) revealed no difference from an age-matched control group, but a highly significant negative correlation between estimates of the net transfer rate (*K_in_^app^*) in the left ventral striatum and individual ACQ scores. In the present study, the regression analysis with the ACQ scores of the alcoholic patients, employing the concurrent and separate estimation of the unidirectional blood-brain clearance (*K*) and the metabolite washout rate (*k_loss_*), revealed a positive correlation with *k_loss_* and a negative correlation with *V_d_*, but no correlation with the magnitude of the clearance (*K*) in left ventral striatum, in which no significant group differences of the three kinetic estimates were detected. We conclude that the earlier finding of a negative correlation between *K_in_^app^* and craving reflects the underestimation of the unidirectional clearance of FDOPA (*K*) in relation to the magnitude of washout of [^18^F]fluorodopamine and its deaminated metabolites (*k_loss_*), which we determined separately from the 120 min PET recordings in the present analysis.

Those patients who experienced the highest craving had relatively low estimates of *V_d_* and high estimates of *k_loss_* in left ventral striatum, predicting a relatively small dopamine pool, characterized by rapid turnover. In functional anatomic terms, the convergence within the ventral striatum of glutamatergic inputs from limbic structures and the dopamine innervation arising in the ventral tegmental region of the mesencephalon is thought to subserve gating of affective signaling from limbic afferents. Given the role of dopamine in incentive salience [34], the finding of decreased [^18^F]fluorodopamine pool and its increased washout in ventral striatum of alcoholic patients may serve to link craving with disinhibition of dopamine turnover.

Chronic alcohol abuse is likely to alter the integrity of dopaminergic pathways in human brain. Indeed, a number of [^11^C]raclopride studies of post-synaptic D_2/3_ receptors revealed reduced binding in striatum of acutely withdrawn alcoholic patients, as compared to healthy control subjects [5], [6], . In contrast, other PET studies with the raclopride congeners [^18^F]fallypride [38] or [^18^F]desmethoxyfallypride ([^18^F]DMFP) [9], [23], and single photon emission computed tomography (SPECT) studies with [^123^I]IBZM [39] or [^123^I]-epidepride [40] revealed no such changes in striatum of acutely abstinent alcoholic patients. Disparate results likewise emerged in the studies with FDOPA cited above, as well as other presynaptic dopamine markers. Thus, the binding of (+)[^11^C]dihydrotetrabenazine to the neuronal vesicular monoamine transporter (VMAT2) was reduced in caudate and putamen of seven males with severe chronic alcohol abuse [41], and an autoradiographic study revealed reduced dopamine transporters in striatum of patients dying with alcoholism [42], as did a SPECT study in patients [40]. In contrast, PET studies with [^11^C]-methylphenidate found no such reduction [36], or an increase in those alcoholic patients with a history of habitual impulsivity and violence [43]. Nonetheless, a follow-up SPECT study showed decreased dopamine transporters in acutely detoxified patients, and a significant increase after prolonged abstinence [44], as also seen in autoradiographic studies of alcohol-preferring vervet monkeys [45]. Thus, there is little consensus for the existence of a presynaptic abnormality in the nigrostriatal pathway of alcoholic patients. On the other hand, down-regulation of presynaptic dopamine markers in alcoholic patients is supported by two reports of attenuated vulnerability of [^11^C]-raclopride binding to psychostimulant challenge in alcoholic patients [5].

The present *V_d_* subtraction maps (Figure 1C,D) and the voxelwise group comparison revealed significantly reduced *V_d_* in the left caudate head of the alcoholic group (Figure 1E), in the absence of any change in *K.* This reduction in dopamine storage capacity was driven by increased *k_loss_*, the rate of washout of [^18^F]fluorodopamine together with its deaminated metabolites. These results in alcoholic patients stand in clear distinction to our earlier findings of increased *K* and decreased *V_d_* estimates in patients with schizophrenia, a circumstance which we termed “poverty in the midst of plenty” [19]. Present finding in alcoholic patients resemble more closely the condition of Parkinson`s disease, except for being restricted to the caudate. Indeed, the reduction of *V_d_* in left caudate nucleus of the present alcoholic group exceeds that seen in patients with early Parkinson’s disease [17] and is equivalent to that seen in three decades of healthy aging [20].

As noted in the introduction, there is no simple physiological interpretation of decreased *V_d_* in the caudate nucleus of abstinent alcoholics, since it may reflect focal degeneration, increased dopamine signaling, or conversely “futile” [^18^F]fluorodopamine synthesis in a cellular compartment containing monoamine oxidase. However, given the preserved estimates of *K*, the present findings are more consistent with altered functional state of dopamine innervations, rather than an actual degenerative process. The caudate nucleus is implicated in cognitive performance pertaining to selection of actions and evaluation of outcomes [46], i.e., the executive functions of the frontal lobe. In healthy control subjects, performance of “prefrontal” cognitive tasks correlated with baseline and pharmacologically stimulated FDOPA utilization in the caudate [47], [48], and had a complex age-dependence with uptake of the alternate dopamine synthesis tracer [^18^F]fluoro-*meta*-tyrosine [49], [50]. Among Parkinson’s disease patients, dopamine transporter availability in the caudate nucleus correlated inversely with sequence learning performance [51], whereas FDOPA utilization correlated inversely with performance of a memory task [52], somatosensory discrimination [53], and the Stroop interference task, as well as executive memory and word fluency [54]–[56]. While the cognitive function of the present patients was not investigated, the findings in caudate are hypothesis-generating for a prospective FDOPA study to test the dependence of executive function on FDOPA dynamics in caudate of detoxified alcoholic patients.

Significant findings of the present study were asymmetric, as noted in earlier PET studies. For example, in [^11^C]raclopride studies of healthy pigs, we noted greater nicotine-evoked binding reductions on the left side [57], and a relationship between novelty seeking trait and amphetamine-evoked binding reductions in the left ventral striatum [58]. In analogous human PET studies, positive subjective effects of amphetamine correlated best with [^11^C]raclopride binding changes on the left side [59], whereas unpredicted monetary reward correlated with [^11^C]raclopride binding changes on the right ventral striatum of healthy males [60]. Although functional asymmetry has been noted in many PET studies of the striatal dopamine innervation, there seems as yet to be no general theory accounting for this phenomenon. In the present context, we speculate that the left-side disturbances in FDOPA metabolism in right-handed alcoholic patients may imply a relationship between cognitive/affective symptoms of alcoholism and hemispheric dominance.

There is a significant correlation between sensation-seeking propensity and differences of extracellular dopamine and dopamine receptors in the striatum [61]. The sometimes impulsive behavior of untreated alcoholics is thought to emerge from an underlying reward deficiency [62]. On the basis of present results, we speculate that individual dynamics of dopamine metabolism in ventral striatum, which would normally be expressed as trait responsiveness to positive stimuli in healthy volunteers, has been subverted into the expression of craving in alcoholic patients. Whereas dopamine release in ventral striatum is implicated in the initial vulnerability to drug seeking [63], dorsal striatum comes to mediate compulsive drug seeking, manifesting as a maladaptive stimulus-response habit, which is triggered and maintained by drug-associated stimuli [64], [65]. The transition from voluntary drug use to loss of control of drug use represents a shift from prefrontal cortical to striatal control over behaviour. This involves a functional shift from ventral striatum, with its connectivity to the prefrontal cortex, to progressive dominance of dorsal, motoric divisions of the striatum [66], [67], by the so-called “spiraling” mechanism mediated by connections through the midbrain dopamine neurons [68], [69]. The primate putamen corresponds to the area of striatum critical for habitual control in rodents [70]. Whereas the putamen is essential for implementation of planned actions, these actions are possibly codified by the caudate [71], where we now present evidence for substantially increased [^18^F]-fluorodopamine turnover in the alcoholic group. However, the putative transition from a vulnerability trait mediated by greater dopamine turnover in the ventral striatum to an acquired state of elevated dopamine turnover in the dorsal striatum might best be investigated in prospective molecular imaging studies of individuals at risk for alcoholism.

### Limitations

In general, comparisons between molecular imaging studies of alcoholism are difficult because of factors such as age, smoking, and other co-morbidities, cumulative alcohol intake, and duration of abstinence. This may explain the neurochemical heterogeneity of type I alcoholics noted in one of the previous PET studies with FDOPA, which reported reduced *K_in_^app^* in the left caudate of two patients [8]. Our present treatment of smoking rate as a nuisance variable in the regression analysis is likely to have removed possible effects of smoking on the group differences in FDOPA kinetic parameters, and their relationship with alcohol craving scores. Indeed, omission of smoking rate from the GLM analysis did not significantly alter the group differences in FDOPA kinetics or their relationship with alcohol craving scores, suggesting that smoking history was not an important factor in the present findings. This stands in contrast from an earlier report in which FDOPA utilization was increased in striatum of smoking subjects [72]. The complex effect of habitual smoking might not completely be untangled by the ANCOVA approach, which is a clear limitation of the present study, even though the patients are not necessarily addicted to nicotine per se. Given the fact that the majority of alcoholics are habitual smokers, it would also be arguable that recruiting alcoholics that prove to have no smoking history may not reflect the clinical reality of the drinking/smoking comorbidity. In general, however, the comorbidity is associated with greater severity of the illness. Considering these complexities of clinical manifestations of alcoholism, there is no single definitive study design. Indeed, numerous study designs could be formulated, and none of them would be fully compelling. Nonetheless, we carefully investigated the potential bias of smoking effect, using the ANCOVA analysis, and found negligible impact from smoking.
